# Manufacturing of recombinant adeno-associated viral vectors: new technologies are welcome

**DOI:** 10.1038/mtm.2015.49

**Published:** 2016-01-06

**Authors:** Eduard Ayuso

**Affiliations:** 1 Atlantic Gene Therapies, INSERM UMR 1089, Université de Nantes, CHU de Nantes, Nantes, France

Recombinant adeno-associated viral vectors (rAAV) are probably the most powerful tools for *in vivo* gene delivery. Encouraging preclinical data have been followed by successful gene therapy clinical trials including Leber’s congenital amaurosis type 2 (refs. 1–3), hemophilia B,^4,5^ and recently choroideremia.^6^ These results together with the market authorization of Glybera, an AAV-based product for the treatment of lipoprotein lipase deficiency,^7,8^ has prompted skeptical investors and biotechnology and pharmaceuticals companies to move into this field. Nonetheless, a major bottleneck to translate these new approaches into the clinic is the manufacturing of rAAV in accordance with current good manufacturing practices (cGMP), requiring costly and timely inefficient protocols. The development of a cGMP-compatible process can be tedious depending on the AAV serotype, vector transgene, and total dose required to launch a phase 1 clinical trial. A recent study by Grieger *et al.*^9^ published in *Molecular Therapy* addresses such challenges for large scale manufacturing of rAAV, providing a flexible protocol based on triple transfection of HEK293 cells in suspension and a purification process that combines ultracentrifugation and ion exchange chromatography. This protocol was validated for multiple serotypes (AAV 1–6, 8, and 9), carrying either single stranded or self-complementary vector genomes with postpurification yields of >10^13^ vector genomes per liter of culture and a purity suitable for clinical use.

The AAV virus belongs to the parvovirus family, specifically the dependoparvovirus subfamily. The members of this subfamily require a helper virus, such as the adenovirus (Ad) or herpes simplex virus, to allow productive infection and replication. The wild-type genome contains three open reading frames encoding for replications proteins (Rep), capsid proteins (Cap) and the assembly activating-protein, and is flanked by two inverted terminal repeats.^10,11^ In 1984, Hermonat and Muzyczka published the first paper of a recombinant AAV vector that was capable of expressing foreign genes in mammalian cells.^12^ Early methods used to produce rAAV vectors relied on transient cotransfection of two plasmids: one carrying the expression cassette of the gene of interest flanked by inverted terminal repeats, and the other carrying the viral *rep* and *cap* sequences. These production protocols utilized auxiliary viruses, like adenoviruses, to provide the helper function. A significant advancement in the field of AAV manufacturing was the identification of Ad regions that mediate AAV vector replication and encapsidation, and their cloning into a helper plasmid that led to the implementation of the triple plasmid transfection method.^13,14^ This system uses the human-derived HEK293 cell line and transiently-transfected three plasmids: (i) rep/cap plasmid, (ii) recombinant vector genome plasmid, and (iii) helper plasmid expressing adenoviral genes. In addition to being a helper virus-free method, the triple transfection protocol offers the flexibility to switch from one serotype to another by simply changing the rep/cap plasmid. For these reasons, triple transfection is widely spread in research grade vector core facilities and has also been used for the manufacturing of clinical grade preparations for phase 1 trials, such as the AAV2 manufactured for Leber Congenital Amurosis trial^15^ or the AAV8 that resulted in long-term correction of hemophilia B patients.^16^ Nevertheless, a limitation of the system is the scalability of the process because HEK293 cells are grown in adherence, and in most of the cases bovine serum is required to obtain good vectors yields.

Grieger *et al.* address the latter challenge by adapting an adherent HEK293 cell line to grow in suspension using serum-free media in shaker flasks and Wave bioreactors up to a 20L-scale. The use of serum-free media is greatly beneficial for cGMP since it increases safety (by eliminating a potential source of adventitious agents contamination), reduces manufacturing costs, and simplifies subsequent purification. Once the cell line was adapted to suspension, Grieger *et al.* have optimized the transfection conditions using a new version of polyethylenimine, named polyethylenimine max, which resulted in transfection efficiencies greater than 70%. Other teams have previously shown that transfection of HEK293 in suspension is possible, however, the transfections efficiencies and production yields in bioreactors of early protocols were significantly lower (<1 × 10^4^ vg/cell)^17^ compared to the yields obtained with this new protocol (10^5^ vg/cell), which resembles the productivity in adherent cells. Although this report represents a significant step forward in the development and implementation of a scalable manufacturing process for AAV, one should caution that the maximal scale tested was only 20 l in Wave bioreactors, which have a limited scalability compared with stirred-tank systems (that can go up to 2,000 l). Preliminary data from Grieger *et al.* indicated that productivity was maintained in 3 l stir tank bioreactors, but “real” scale up (>200 l) has yet to be demonstrated.

The vector assembly (upstream) methodology based on triple transfection is one possible strategy, but other competing technologies have already demonstrated enormous potential for large scale production (>200 l) (review in ref. 18). Insect cells from *Sodoptera frugiperda* (such as *Sf9*) are cultured in suspension with serum-free medium and can be infected by baculovirus expression vectors derived from *Autographa californica nucleopolyhedrovirus* to provide rep/cap genes and rAAV vector genomes.^19^ Multiple optimizations from R. Kotin’s team led to the development of a process with the capacity to produce >10^16^ vg from 200 l stirred-tank bioreactors.^20^ The baculovirus expression vectors/Sf9 platform was also used by uniQure for the manufacturing of Glybera (AAV1) to conduct their phase 3 trial and obtain market authorization. An alternative upstream process with demonstrated feasibility at large scale (>200 l) is the use of mammalian-derived producer cell lines containing the rep/cap genes and the AAV vector integrated in the genome. In this case, the amplification of the rAAVs is initiated upon infection by a helper virus, such as Ad.^21^ This system was used to produce the AAV1-SERCA2a vector used for phase 2b trials for advanced heart failure^22^ and the upstream process was designed to be compatible with bioreactors of 2,000 l as announced by the partnership between Celladon and Novasep. Another viable approach for rAAV manufacturing consists of using a recombinant herpes simplex virus complementation system in suspension-cultured baby hamster kidney (sBHK) cells. Two recombinant herpes simplex virus helper viruses, one containing the rep/cap genes and the other containing the rAAV vector genome are used to coinfect sBHK cells grown in serum-free medium.^23^ rAAV vectors manufactured under cGMP conditions using this method have been used for a phase 2 clinical trial for expression of α1-antitrypsin.^24^ Thus, it is evident that different upstream technologies are able to deliver cGMP material in sufficient quantities to support phase 1/2 trials.

For the purification (downstream) process, Grieger *et al*. incorporate two major steps upon clarification, the ultracentrifugation of rAAV particles in a Iodixanol gradient followed by anion exchange chromatography (Q Hyper-D columns or HiTrapQ HP columns). This protocol resulted in highly pure vector stocks with minimal empty capsids contamination (below 20%) and showed similar performance for many serotypes (1–6, 8, and 9). The universality of the method is remarkable, and the removal of empty capsids is a critical point to reduce immune responses due to capsid antigens.^25^ Nonetheless, the scalability of the ultracentrifugation step is a caveat since it can accommodate only a few containers per run. While the authors have successfully purified up to 5 × 10^14^ vg from a 20 l bioreactor by ultracentrifugation, it is not clear how it will be possible to accommodate larger amounts of vectors (for instance >10^16^ vg). As shown by Cecchini and colleagues,^20^ purification protocols based on a single immunoaffinity chromatographic column can produce >10^16^ vg per run and are ideal in terms of industrialization, but unfortunately these columns are not able to discriminate between empty and full particles. The use of ion exchange columns have the advantage of being adaptable to large scale-industrial processes and, under certain conditions, can be used to remove empty particles.^26^ However, the purification protocols based on ion exchange columns require a significant amount of process development and likely has to be adapted for each AAV serotype, being very sensitive to changes in the upstream biomass (pH, cell densities, media composition, etc.).

In summary, the system described by Grieger *et al.* is a highly flexible platform for manufacturing rAAV intended for first-in-man use because of the low production times and high purity of the product. Furthermore, the authors investigated if their system could be adapted to reduce the high costs that arise from manufacturing of GMP source plasmid for large-scale transfection. For those serotypes that are efficiently secreted into the media, Grieger *et al.* demonstrated that several rAAV harvests could be done at different time-points from the same transfected cells. The later approach resulted in an increase of up to sixfold in vector productivity, reducing in turn the cost of plasmid material and cell culture reagents. However, scalability and industrialization of the whole process is not yet solved and other technologies, such as the baculovirus expression vectors/Sf9 or mammalian-derived producer cell lines have shown clear advantages in this regard.

Given these considerations, how should one choose between one or another technology if all of them are able to deliver cGMP compatible vectors? The answer could depend on for the costs and duration of manufacturing and the quality of the vector product. While cost and the time are objective parameters, determining the quality of a rAAV stocks is not straightforward because vector analytics are not standardized, and contaminants that are present could be radically different depending on the process (residual helper virus versus residual plasmid sequences, human cells versus insect cells versus animal cells, etc.). Comparative studies between all production platforms should be conducted rigorously and, ideally, performed by independent laboratories using complementary analytical methods. Advances in vector manufacturing are crucial for the success of human gene therapy. The future will show whether a “wining platform” has now been established or the best approach has yet to come. New technologies are always welcome.

## Notes

The author declared no conflict of interest
